# Cycloplegic refraction in children

**Published:** 2024-05-15

**Authors:** May Ho, Priya Morjaria

**Affiliations:** 1 Optometry and Primary Care Adviser: The Fred Hollows Foundation, Melbourne, Australia.; 2 Assistant Professor and Public Health Optometrist: London School of Hygiene & Tropical Medicine and Head of Global Programme Design: Peek Vision, UK.

**Cycloplegic refraction makes it possible to accurately measure a child's refractive error and provide appropriate correction.**

**Figure:**
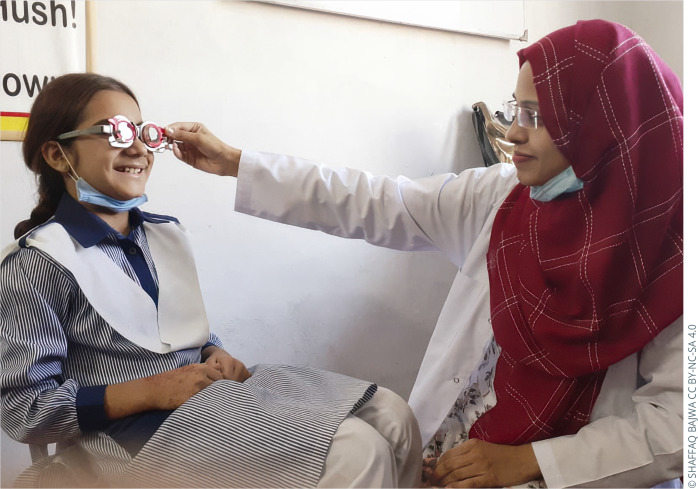
Children are eager to please and they may try too hard to give the ‘correct’ answers during refraction; this can result in an inaccurate spectacle prescription. pakistan

The prevalence of uncorrected refractive errors in children is on the rise globally.^1^ Uncorrected refractive errors can affect children's ability to develop good vision and also hamper their education and social activities. It is therefore important to measure a child's refractive errors accurately.

Accommodation is the ability of the eye to focus on objects at varying distances. This ability is greatest in children and declines with age – usually becoming noticeably reduced after the age of 40 with the development of presbyopia, when the eye loses the ability to focus on near objects.

Children's wonderful ability to accommodate can, however, affect the accuracy of refractive error assessment: children often try too hard to give the ‘correct’ answers, which leads them to subconsciously over-focus, resulting in an inaccurate spectacle prescription. Typically, myopia will be overcorrected, and hyperopia (long-sightedness) will be under-corrected; astigmatism could also be misdiagnosed. Children could therefore be prescribed unnecessary spectacles for myopia or astigmatism, or miss out on getting spectacles to correct hyperopia. Wearing incorrectly prescribed spectacles could lead to non-optimal vision, eye strain, headaches, nausea, strabismus (squint), and amblyopia (lazy eyes), and it could even hasten myopia progression.^2^

In order to achieve accurate results, accommodation can be controlled by using cycloplegic eye drops to temporarily paralyse the muscles used in accommodation, followed by retinoscopy (known as wet retinoscopy) or subjective refraction (wet refraction).

Commonly used eye drops for this purpose are cyclopentolate, tropicamide, and atropine.

**Cyclopentolate 1%** (0.5% for children aged less than 1 year) is accepted as the gold standard for cycloplegic refraction. It is fast and relatively short acting and is suitable for most patients.

**Tropicamide 1%** is fast acting and has a shorter duration but has a weaker cycloplegic effect, so it is better suited to older children. While tropicamide has been shown to yield similar refraction results to cyclopentolate, care needs to be taken in cases of high hyperopia, strabismus, or when there is inconsistency between examination results and clinical manifestations of vision problems.^3^

“Children's wonderful ability to accommodate can, however, affect the accuracy of refractive error assessment.”

Both types of drops are safe, with cyclopentolate reporting some extremely rare side effects.^4^ Systemic absorption can be minimised by occluding the puncta after instillation and wiping away any excess drops. Where cyclopentolate is not available, or in children with known sensitivity, central nervous system disorders, or Down syndrome, tropicamide is a viable alternative.^5^

**Atropine 1%** is commonly used in some settings, but caution is advised. Atropine is slow acting and has a longer duration with effects lasting several days; this can cause discomfort. In children with very darkly pigmented eyes, atropine is often recommended when cyclopentolate is not effective. Note that atropine can be poisonous when absorbed systemically: a 3 g tube of 1% atropine ointment can be fatal if ingested accidentally by a small child.^6^

## When is cycloplegic refraction needed?

Ideally, all children with suspected refractive errors should undergo cycloplegic refraction at least once when they are identified, and subsequently when it is suspected that the child is accommodating during refraction. However, there are legislative limitations in many countries as to who can use pharmaceutical agents for eye examinations, with the use of diagnostic eye drops often restricted to medical doctors only.

Where the use of pharmaceutical agents is not allowed, alternative methods of controlling accommodation (such as fogging lenses for retinoscopy or refraction) may be considered, although these may be less effective^7^,^8^ In older children (aged 11–18 years), conducting retinoscopy without cycloplegia (dry retinoscopy) may provide satisfactory results. However, if any of the indications listed in the panel are present, and dry refraction does not yield reliable results, then cycloplegic refraction is indicated.

## How to perform cycloplegic refraction in school-aged children

Children often do not like drops in their eyes, and cycloplegic drops can be particularly uncomfortable.

Although some eye care providers suggest instilling an anaesthetic eye drop before instilling the cycloplegic eye drop, in our experience instilling a single drop (just the cycloplegic agent) is less stressful for the child.

The following steps are designed to be reassuring for the child and to support their cooperation with the process of cycloplegic refraction.

**Figure 1:**
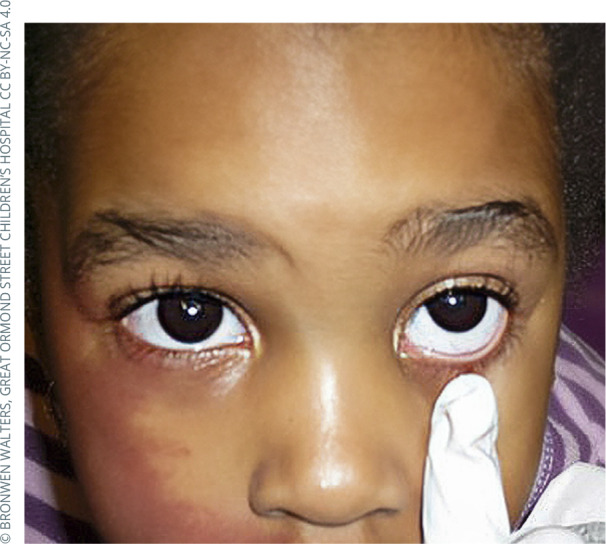
Gently pull down the lower eyelid to create a ‘sac’. uk

1. Wash your hands and explain to the child and/or their caregiver what you are going to do. We recommend telling the child that the drops may make their eyes feel ‘funny’; avoid using the word ‘sting.’
2. Ask the child to sit on their parent or carer's lap; this is often the most comfortable place for the child.
3. Ask the child to look up. As shown in Figure 1, gently pull the lower eyelid down to create a ‘sac’ (the lower fornix). Instil **one** drop of cyclopentolate 1% (or tropicamide 1%, if cyclopentolate is not available) into the lower fornix.
4. Wipe away any excess drops and gently press a finger against the inner corner of the eye, next to the nose, to occlude the puncta for 1–2 minutes. This will minimise systemic absorption.
5. Repeat steps 3 and 4 in the other eye.
6. If, after 15 minutes of instilling cyclopentolate, the pupils do not appear to be dilating, **and** the child has darkly pigmented eyes, instil another drop and repeat punctal occlusion for 1–2 minutes.
7. Wait 30–40 minutes (15–25 minutes for tropicamide 1%), then check that the pupils are dilated and do not constrict when light from a torch is shone onto them. If the pupils constrict, wait another 5 minutes and check reactivity to light again.
8. When the pupils are unreactive to light, perform retinoscopy or subjective refraction.
9. Advise parents that the child will have dilated pupils, blurry vision up close, and sensitivity to bright light for several hours afterwards.

For more practical tips on instilling eye drops in a young child, visit tinyurl.com/CEHJ-eyedrops.

**Figure 2:**
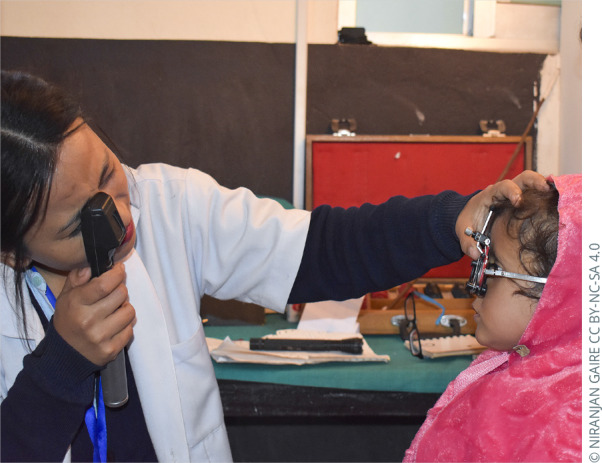
Wet retinoscopy (after instilling cycloplegic eye drops) can help you to achieve more accurate refraction results in younger children who have active accommodation. nepal

## A need for advocacy

Atropine and tropicamide are widely approved for use in low- and middle-income settings. Cyclopentolate is less widely approved, but it is listed as an alternative to atropine and tropicamide in the WHO Model List of Essential Medicines 23rd List (2023).

To address the increasing demand for accurate refraction in children, we need to advocate for legislation that would allow qualified and appropriately trained eye health professionals, such as optometrists, to use the best cycloplegic agent (cyclopentolate). This is especially important given the increased prevalence of refractive errors in children, particularly myopia.

**Other indications for cycloplegic refraction**

Cycloplegic refraction can be helpful in the following circumstances. Refer to your professional association's guidelines for more detailed recommendations.

- Undiagnosed manifest esotropia
- An esotropia recognised by the parent or carer
- Unstable or uncompensated esophoria
- Significant risk factors for esotropia or amblyopia, such as family history or high refractive errors
- Good visual acuity is not achieved following a dry refraction
- Stereoscopic acuity is poor
- Latent hyperopia or pseudomyopia is suspected.
